# Associations of fetal growth patterns and growth in the first two years of life: a longitudinal study of a Beijing birth cohort

**DOI:** 10.1080/07853890.2026.2757376

**Published:** 2026-11-02

**Authors:** Xiangli Meng, Xiaofei Li, Lina Yi, Xiaowei Xiong, Shangjin Zhong, Minghui Wu, Na Zhang, Qingqing Wu

**Affiliations:** Beijing Obstetrics and Gynecology Hospital, Capital Medical University, Beijing Maternal and Child Health Care Hospital, Beijing, China

**Keywords:** Fetal growth pattern, child growth, group-based trajectory model

## Abstract

**Background:**

This study aimed to evaluate the association between fetal growth patterns and early childhood growth rate.

**Methods:**

In total, 6,370 mother-child pairs were included in a birth cohort in Beijing. Fetal biometric parameters were measured at 20–24, 28–32, 33–36 weeks, and at ≥37 weeks of gestation, and infant weight and length were assessed from birth to 24 months of age. Changes in the weight-for-length Z-score (WLZ) from birth to 3, 6, 9, 12, 18, and 24 months of age were used to classify infant growth velocity as slow (change in WLZ < −0.67) and or rapid (change in WLZ >0.67). A group-based trajectory model was used to identify fetal growth patterns. Generalised linear models and longitudinal modified Poisson GEE models were used to assess the association between fetal trajectories and infant growth velocity.

**Results:**

Five fetal growth patterns were identified. Early acceleration with late decline and a moderate final Z score (EALD-M) showed no obvious trend in growth rate from birth to 24 months of age. Early deceleration (ED) was significantly associated with an increased risk of rapid growth at 3, 6, 9, 12, 18, and 24 months (risk ratio [RR] = 1.93–2.56), followed by stable growth (SG) (RR = 1.47–1.74). Early acceleration with late decline and high final Z score (EALD-H) was associated with an increased risk of slow growth at 3, 6, 9, 12, 18, and 24 months (RR = 1.64–2.02), followed by late acceleration growth (LA) (RR = 1.26–1.48).

**Conclusion:**

ED and SG are associated with rapid growth, whereas LA and EALD-H are associated with slow growth.

## Introduction

Being underweight or obese is associated with adverse health outcomes. Recently, the prevalence of childhood overweight and obesity has shown an overall upward trend [1]. According to the Chinese National Survey on Students’ Constitution and Health, the prevalence of obesity among children and adolescents aged 7–18 years in China increased from 0.1% in 1985 to 9.6% in 2019 [2]. The rapidly increasing prevalence of obesity among children has increased the health burden. Rapid growth in the first two years of life is associated with elevated odds of later becoming overweight and obesity [3–6]. Furthermore, rapid growth is linked to adverse metabolic outcomes, including insulin resistance, hypertension, and early onset cardiovascular disease [7,8]. Collectively, these findings highlight that the “first 1,000 days” represents a critical window for the prevention of chronic diseases across the lifespan.

The uterine environment and early growth are critical for future body weight [9]. A growing body of research has identified the fetal period as a critical window for biological and behavioural plasticity linked to the risk of overweight and obesity [10,11]. High and low birth weights and rapid growth in the first two years after birth are associated with an increased risk of overweight and obesity [5,12–14]. Intra- and extrauterine growth are often treated as distinct processes, with limited longitudinal research investigating the association between fetal and infant growth trends. Although evidence indicates that birth weight and infant growth are linked to overweight and fat mass in later childhood [10,15], whether infants with similar birth weights but divergent in utero growth trajectories differ in postnatal growth over the first two years remains unclear.

Early fetal growth trajectories may reshape fetal lipid metabolic programming and modulate steroid metabolism and hormone synthesis, which constitute key mechanisms of fetal programming that regulate fetal body composition and affect postnatal growth velocity [16,17]. Thus, we propose that fetal growth patterns, defined as the pattern of change in estimated fetal weight (EFW) Z-scores during pregnancy, can provide critical insights into the growth rate, defined as the change in weight for length Z-scores (WLZ) for infants aged 0–2 years, from birth to a specific age. Integrating longitudinal fetal biometric parameters into machine learning algorithms allows for the dynamic tracking of fetal growth and development and identification of potential heterogeneity, which is useful for monitoring fetal growth patterns [18,19]. This study used (1)group-based trajectory modelling (GBTM) approach to identify foetuses with similar growth trajectories and establish growth patterns based on the trajectory of EFW Z-score changes and (2)analysed the association between each pattern subgroup and growth velocity at 3, 6, 9, 12, 18 and 24 months of age.

## Materials and methods

### Study population

This study included singleton pregnant women who received prenatal care and delivered at the BJOG between 1 February 2018 and 31 March 2021. Data analysis was conducted between November 2025 and February 2026. This study enrolled women who received prenatal care before 14 weeks of gestation. Gestational age was determined using crown–rump length measurement [20], except in cases of assisted reproduction, for which the embryo transfer was known. Infants born preterm were assessed using the corrected age. The length and weight were measured from birth to 24 months of age. Participants were eligible for enrolment (1) if they had singleton pregnancy, (2) at least three fetal biometric measurements available (between 20–24, 28–32, 33–36, and ≥37 weeks of gestation, corresponding to routine ultrasonography periods in clinical practice in China), (3)had at least one additional anthropometric measurement available at 3, 6, 9, 12, 18, and 24 months of age. Participants meeting any of the following criteria were excluded: (1) maternal age <18 years, (2) multiple pregnancy, (3) <2 fetal biometric measurements, (4) no anthropometric data available for the infant between 0–2 years, were excluded. The Beijing birth cohort comprised 6,592 participants from 2018–2021. Of the 6,592 mother-child pairs, 6,558 were singleton births. There were 188 exclusions due to missing WLZ measurements between birth and 24 months of age. Finally, 6,370 infants had a WLZ measure at birth and at least one measure at 3,6, 9, 12, 18 or 24 months of age, constituting the study sample (Figure 1). The delivery and newborn characteristics of the children lost to follow-up were not significantly different from those of the children included in the final cohort (data available upon request).

**Figure 1.**
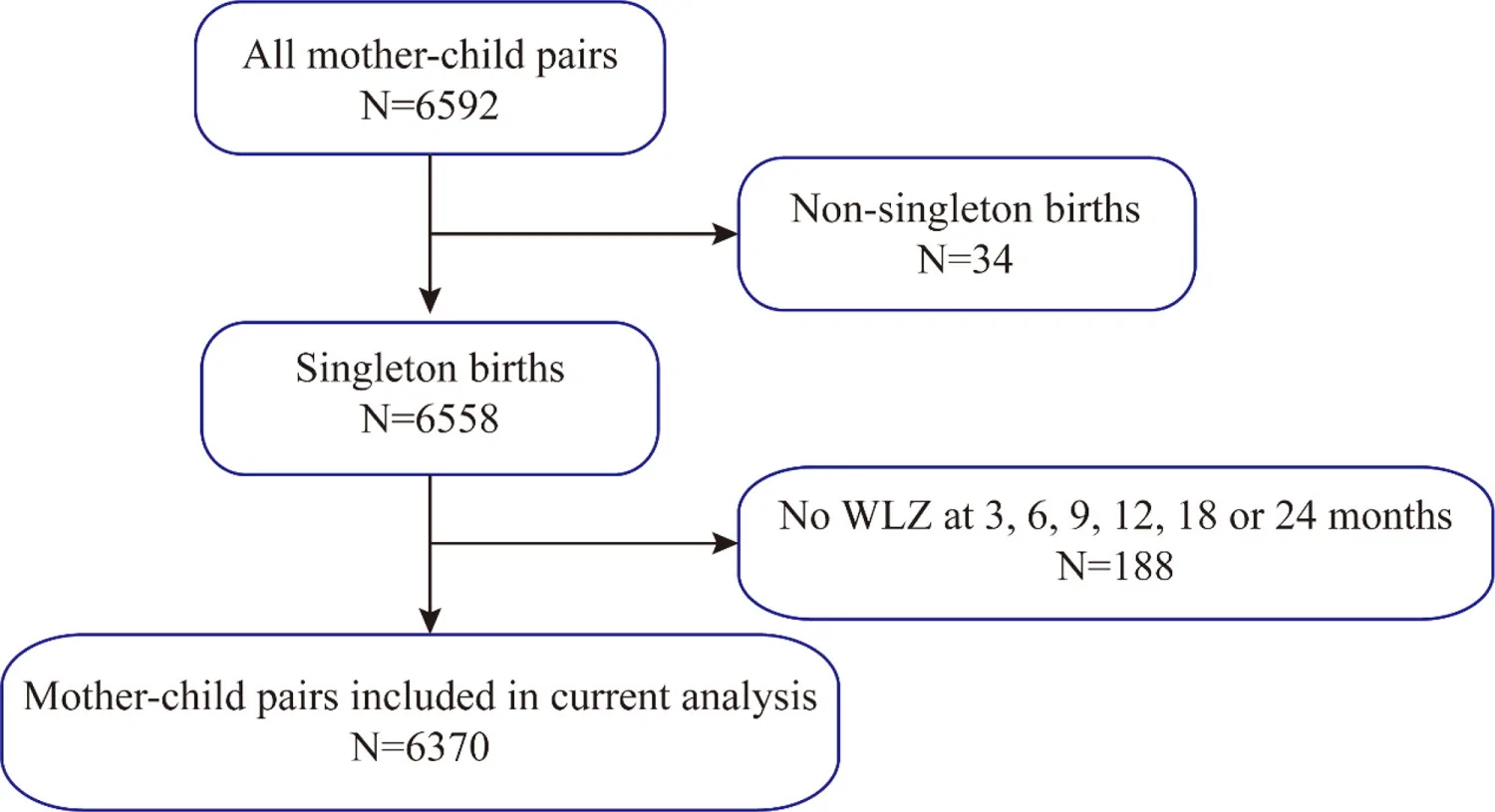
Flowchart of participant inclusion. WLZ: weight-for-length Z-score.

## Fetal biometry measurements during pregnancy

The primary exposure was the fetal growth pattern. Fetal biometric parameters (head circumference [HC], abdominal circumference [AC], and femur length [FL]) were extracted from medical records using measurement methods following ISUOG guidelines [21]. Fetal biometric measurements were grouped into timing categories based on gestational age during ultrasound: 20–24, 28–32, 33–36, and ≥37 weeks, with at least four weeks between each measurement. EFW was calculated using the AC, HC, and FL with the Hadlock-3 formula and converted into gestational age-specific Z-scores using the INTERGROWTH-21st standards [22,23]. The GBTM was used to identify clusters based on longitudinal EFW Z-scores.

## Child growth

Infant weight and length data from birth to 24 months of age were collected using standard anthropometric methods during routine physical examinations [24]. The study included only data from infants with anthropometric measurements at 3 months (2–4 months), 6 months (5–7 months), 9 months (8–10 months), 12 months (11–13 months), 18 months (17–19 months), and 24 months (23–25 months). Continuous outcomes, including length-for-age Z-score (LAZ), weight-for-age Z-score (WAZ), and weight-for-length Z-score (WLZ), were calculated and expressed using World Health Organization (WHO) Child Growth Standards Z-scores [25]. Infants were classified as SGA (<90th percentile weight-for-gestational age), appropriate-for-gestational-age (AGA; 10–90th percentile weight-for-gestational age), or large-for-gestational-age (LGA; >90th percentile weight-for-gestational age) based on birth weight and gestational age using the INTERGROWTH-21st standards [24]. The changes in WLZ at 3, 6, 9, 12, 18, and 24 months of age relative to birth were used to define growth velocity at each of these target age points; slow growth (WLZ score difference; < −0.67), reference range (WLZ score difference; −0.67–0.67), and rapid growth (WLZ score difference; >0.67) [26].

## Covariate assessment

At the first prenatal visit, participants completed a detailed questionnaire assessing their demographics (age, education, height, and weight) and medical history (previous pregnancies and pregnancy complications). Data on maternal complications during pregnancy (including diabetes and hypertensive disorders of pregnancy [HDP, including gestational hypertension and preeclampsia]) were extracted from electronic health records. According to the WHO classification [27], patients are classified as underweight (body mass index [BMI]; <18.5 kg/m^2^), normal weight (BMI; 18.5–24.9 kg/m^2^), overweight (BMI; 25–29.9 kg/m^2^), or obese (BMI; ≥30 kg/m^2^). These WHO adult BMI thresholds exclusively apply to the classification of parental weight. Maternal gestational weight gain (GWG) was calculated as the difference between the weight measured before childbirth and self-reported pre-pregnancy weight. GWG was categorised as inadequate, recommended, or excessive according to the Institute of Medicine classification [28].

Birth outcomes were extracted from postdelivery medical records, including gestational age at birth, sex, birth length, birth weight, mode of delivery, placental weight, placental morphology, and umbilical cord length. Gestational age-specific Z-scores for birth weight and length were expressed using the Z-score standards of INTERGROWTH-21st [24]. The study design is shown in eFigure 1 in Supplement 1.

## Statistical analysis

In this study, the GBTM was applied to construct trajectory models [29] and identify clusters based on longitudinal records of EFW Z-scores. The EFW Z-score of each foetus at 10 weeks of gestation was set to 0 to standardise the starting point, enabling the GBTM to focus on identifying distinct patterns of change in fetal growth. The final model was selected based on the lowest Bayesian Information Criterion, posterior probabilities ≥0.7, and clinical interpretability. Parameters were estimated *via* maximum likelihood estimation, and each foetus was assigned to the cluster with the highest posterior probability; cluster assignments were derived using the “PROC TRAJ” procedure in SAS software. Alluvial plots were used to illustrate the transitions between fetal growth trajectories and the traditional classification based on birth weight categories. Linear regression models were used to evaluate the relationship between the growth trajectories and rate from birth to 24 months. The LSMEANS option in the SAS PROC MIXED procedure was used to perform difference tests and calculate the least-squares means of fixed effects to predict the population marginal means.

Continuous variables with a normal distribution were presented as mean ± SD, whereas non-normally distributed variables were presented as median (IQR). One-way ANOVA or Kruskal–Wallis tests were used for continuous variables. Categorical variables are presented as percentages and were analysed using Chi-square or Fisher’s exact tests. We used multivariable Poisson regression models with a robust variance estimator to estimate the risk ratios (RRs) and associated 95% confidence intervals (CIs) for fetal growth rate. Confounders were identified a priori according to literature and directed acyclic graphs (eFigure 2 in Supplement 1).

Missing data ranged from 0% for maternal age and education, HDP, sex, and gestational age at delivery to 0.5% for the placental (eTable 1 in Supplement 1). We used multiple imputation using chained equations with 20 imputations the missing values. We performed stratified analyses according to maternal education, maternal pre-pregnancy BMI ≥25 kg/m^2^, HDP (yes or no) and diabetes during pregnancy (yes or no). All analyses were performed using SAS version 9.4.

## Results

### Basic characteristics

The participants’ mean age was 32.9 years. Most were nulliparous (69.4%) with a mean pre-pregnancy BMI of 21.9 kg/m^2^ and the average GWG of 13.0 kg. Spontaneous vaginal delivery was recorded in 4,114 participants (64.6%), with a mean gestational age at delivery of 38.9 weeks. The sex ratio of the newborns was balanced (52.5% males and 47.5% females) with a mean birth weight of 3.35 kg (eTable 2 in Supplement 1).

### Fetal growth pattern

We determined the trajectory pattern based on EFW Z-scores measured at multiple time points. The optimal model comprised a five-class latent growth model (eTable 3–4 in Supplement 1). The growth trajectories of EFW Z-scores for the five clusters are shown in Figure 2(A). Dot plots of the individual fetal measurements for each growth pattern are provided in eFigure 3 in Supplement 1. Based on the pattern of longitudinal EFW Z-score curves, we categorised the trajectories as groups [1]: early deceleration growth pattern (ED, 403 [6.3%]) [2]; stable growth pattern (SG, 2,353 [36.9%]) [3]; late acceleration growth pattern (LA, 1,256 [19.7%]) [4]; early acceleration with late decline and moderate final Z-score growth pattern (EALD-M, 1,573 [24.7%]); and [5] early acceleration with late decline and high final Z-score growth pattern (EALD-H, 785 [12.3%]).

**Figure 2.**
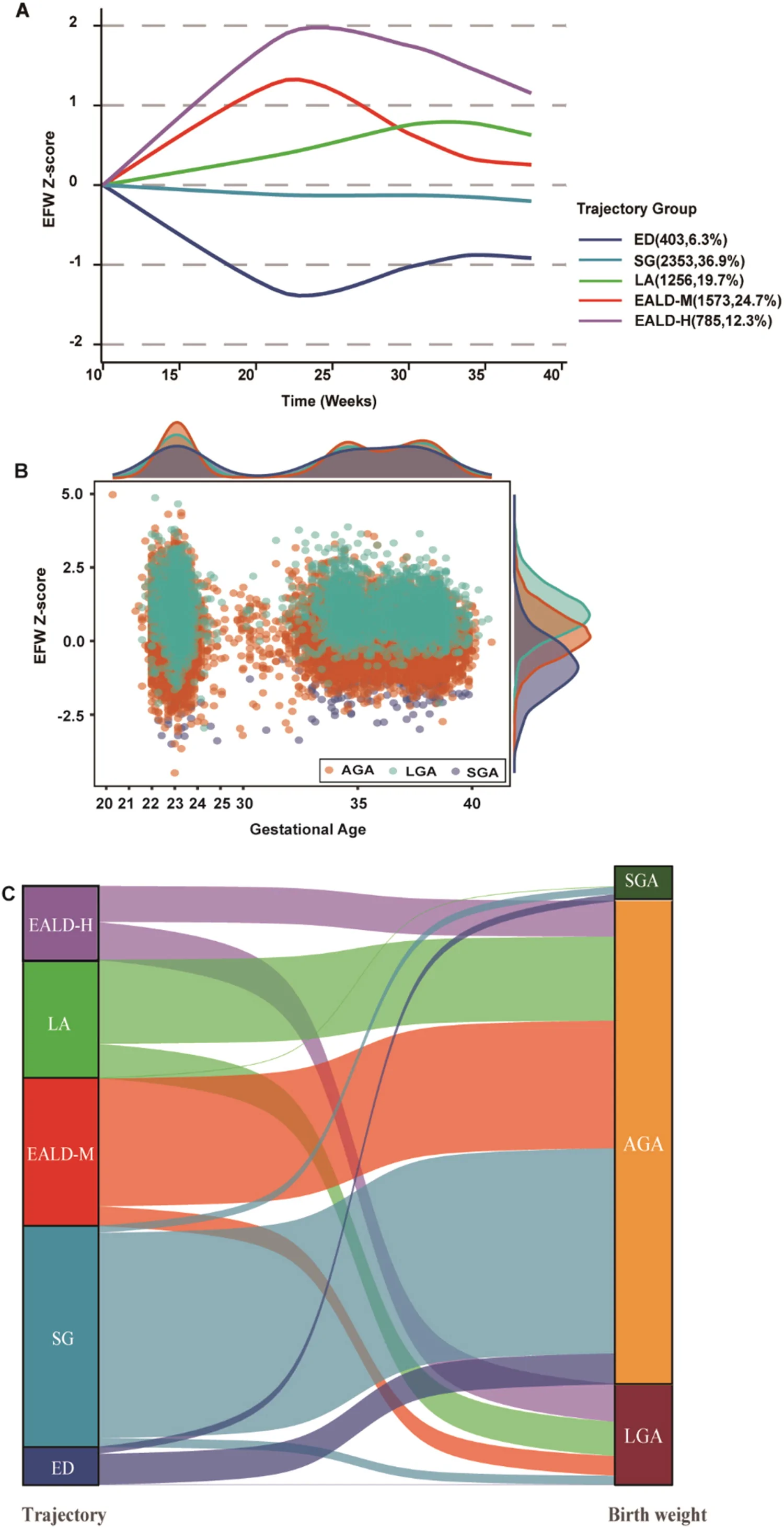
EFW growth patterns according to GBTM and categories based on birth weight and gestational age. (A) The EFW growth patterns in five groups derived from longitudinal records of EFW Z-scores. Group sizes are presented as percentages. Trajectory groups: ED, Early deceleration growth; SG, Stable growth; LA, Late acceleration growth; EALD-M, Early acceleration with late decline and moderate final Z-score growth; EALD-H, Early acceleration with late decline and high final Z-score growth. (B) The EFW Z-scores according to categories based on birth weight and gestational age (AGA, LGA, SGA). Group sizes are presented as percentages. (C) Alluvial plot shows the transitions of classifications for individual fetus between group-based trajectory and birth weight categories. Abbreviations: EFW: estimated fetal weight; GBTM: group-based trajectory modelling; AGA: appropriate-for-gestational-age; LGA: large-for-gestational-age; SGA: small-for-gestational-age.

Classification by birth weight and gestational age showed that 5,141 neonates (80.7%) met the criteria for AGA, 157 for SGA (2.5%), and 1,072 (16.8%) for LGA. The changes in EFW Z-scores for SGA, LGA, and AGA are plotted in Figure 2(B). The EFW growth trends were challenging to distinguish among the three groups. Fetal transitions between trajectories and birth weight classifications are shown in Figure 2(C) and eTable 5 in Supplement 1. Overall, the growth trajectories were heterogeneous across the AGA, SGA, and LGA groups.

### Association of trajectories of fetal growth and child growth

Table 1 presents the baseline characteristics of the pregnant women across the subgroups stratified by fetal growth patterns. The ED pattern had the youngest maternal age (32.2 years) and lowest pre-pregnancy BMI (21.1 ± 3.1 kg/m^2^), along with elevated proportions of underweight (17.87%), inadequate GWG (33.75%), primiparity (75.68%), assisted reproduction (7.20%), thrombophilia (5.21%), and HDP (12.66%). The SG pattern was characterised by an increased proportion of women with inadequate GWG (34.46%) and primiparity (71.87%). In the EALD-M pattern, higher proportions of normal pre-pregnancy BMI (74.13%), normal GWG (45.07%), and normal blood pressure (93.39%) were observed. The LA pattern had elevated proportions of pre-pregnancy overweight and obesity (19.42%) and excessive GWG (28.34%). The EALD-H pattern featured the oldest maternal age (33.8 years, *p* < 0.01), highest pre-pregnancy BMI (22.8 ± 3.3 kg/m^2^), and greatest GWG (13.4 ± 4.6 kg), with increased proportions of maternal age ≥35 years (41.40%), excessive GWG (28.41%), pre-pregnancy overweight and obesity (21.91%) and anaemia (30.19%). Comparisons of delivery and neonatal data across subgroups stratified by fetal growth patterns are presented in eTable 6 in Supplement 1.

**Table 1.** Maternal characteristics of pregnancies in trajectory-based subgroups.

Variables	ED (*n* = 403)	SG (*n* = 2353)	LA (*n* = 1256)	EALD-M (*n* = 1573)	EALD-H (*n* = 785)	*P*
Maternal age (SD), y	32.2(4.0)	32.5(3.9)	32.9(3.9)^ab^	33.0(4.1)^ab^	33.8(4.3)^abcd^	<0.001
Maternal age category						<0.001
<35	291 (72.21)	1688 (71.86)	843 (67.12)	1042 (66.28)	460 (58.60)	
≥35	112 (27.79)	661 (28.14)	413 (32.88)	530 (33.72)	325 (41.40)	
Paternal age	32.57 ± 4.82	32.47 ± 4.55	32.97 ± 4.58	32.90 ± 4.94	33.72 ± 4.98	<0.001
Maternal prepregnancy BMI	21.1 ± 3.1	21.4 ± 3.1	22.3 ± 3.4^ab^	21.9 ± 3.4^abc^	22.8 ± 3.3^abcd^	<0.001
Maternal prepregnancy BMI category						<0.001
18.5–24.9	283 (70.22)	1730 (73.52)	889 (70.78)	1166 (74.13)	563 (71.72)	
<18.5	72 (17.87)	346 (14.70)	123 (9.79)	178 (11.32)	50 (6.37)	
25–29.9	44 (10.92)	230 (9.77)	197 (15.68)	192 (12.21)	146 (18.60)	
≥30	4 (0.99)	47 (1.98)	47 (3.74)	37 (2.35)	26 (3.31)	
Paternal BMI	24.79 ± 3.44	24.59 ± 3.35	24.81 ± 3.22	24.75 ± 3.16	25.09 ± 3.24	0.043
Paternal BMI category						0.015
18.5–24.9	224(55.58)	1347(57.25)	679(54.06)	876(55.69)	399(50.83)	
<18.5	12(2.98)	50(2.12)	24(1.91)	23(1.46)	7(0.89)	
25–29.9	141(34.99)	797(33.87)	464(36.94)	579(36.81)	324(41.27)	
≥30	26(6.45)	159(6.76)	89(7.09)	95(6.04)	55(7.01)	
Maternal education						0.120
Junior high school or below	26(6.45)	94(3.99)	60(4.78)	86(5.47)	41(5.22)	
Bachelor’s degree or Junior college	279(69.23)	1599(67.96)	879(69.98)	1094(69.55)	536(68.28)	
Master’s degree or above	98(24.32)	660(28.05)	317(25.24)	393(24.98)	208(26.50)	
Paternal education						0.018
Junior high school or below	26(6.45)	127(5.40)	86(6.85)	89(5.66)	64(8.15)	
Bachelor’s degree or Junior college	294(72.95)	1572(66.81)	837(66.64)	1072(68.15)	522(66.50)	
Master’s degree or above	83(20.60)	654(27.79)	333(26.51)	412(26.19)	199(25.35)	
Maternal Nation						0.772
Han	385(95.53)	2252(95.71)	1208(96.18)	1498(95.23)	754(96.05)	
Other nation	18(4.47)	101(4.29)	48(3.82)	75(4.77)	31(3.95)	
GWG	13.3 ± 5.1	12.8 ± 4.4	13.2 ± 4.4	12.9 ± 4.5	13.4 ± 4.6	0.011
GWG category						<0.001
Recommended	161(39.95)	1051(44.67)	556(44.27)	709(45.07)	345(43.95)	
Excessive	106(26.30)	491(20.87)	356(28.34)	347(22.06)	223(28.41)	
Inadequate	136(33.75)	811(34.46)	344(27.39)	517(32.87)	217(27.64)	
Conception						0.021
ART	29 (7.20)	111 (4.72)	72 (5.73)	94 (5.98)	60 (7.64)	
Natural	374 (92.80)	2242 (95.28)	1184 (94.27)	1479 (94.02)	725 (92.36)	
Uniparous						<0.001
No	98(24.32)	662(28.13)	408(32.48)	482(30.64)	298(37.96)	
Yes	305(75.68)	1691(71.87)	848(67.52)	1091(69.36)	487(62.04)	
Parity						<0.001
0 time	305(75.68)	1691(71.87)	848(67.52)	1091(69.36)	487(62.04)	
1 times	95(23.57)	630(26.77)	395(31.45)	461(29.31)	287(36.56)	
≥2 times	3(0.74)	32(1.36)	13(1.04)	21(1.34)	11(1.40)	
Diabetes during pregnancy						0.161
No	342(84.86)	1992(84.66)	1039(82.72)	1329(84.49)	639(81.40)	
Yes	61(15.14)	361(15.34)	217(17.23)	244(15.51)	146(15.60)	
Hypertension disorder in pregnancy						0.001
No	352 (87.34)	2172 (92.31)	1170 (93.15)	1469 (93.39)	718 (91.46)	
Yes	51 (12.66)	181 (7.69)	86 (6.85)	104 (6.61)	67 (8.54)	
Anemia						<0.001
No	327(81.14)	1886(80.15)	925(73.65)	1233(78.39)	548(69.81)	
Mild anemia	61(15.14)	358(15.21)	260(20.70)	272(17.29)	183(23.31)	
Moderate or severe anemia	15(3.72)	109(4.63)	71(5.65)	68(4.32)	54(6.88)	
Thrombophilia						<0.001
No	382(94.79)	2331(99.07)	1242(98.89)	1560(99.17)	778(99.11)	
Yes	21(5.21)	22(0.93)	14(1.11)	13(0.83)	7(0.89)	

Trajectory subgroups: ED: early deceleration growth; SG: stable growth; LA: late acceleration growth; EALD-M: early acceleration with late decline and moderate final Z-score growth; EALD-H: early acceleration with late decline and high final Z-score growth. BMI: body mass index (calculated as weight in kilograms divided by height in meters squared); GWG: gestational weight gain (calculated as the difference between the weight measured before childbirth and self-reported prepregnancy weight); ART: assisted reproductive technology.

a, compared with the ED phenotype.

b, compared with the SG phenotype.

c, compared with the LA phenotype.

d, compared with the EALD-M phenotype.

Data on LAZ, WAZ, and WLZ at birth and 3, 6, 9, 12, 18, and 24 months of age in infants across subgroups stratified by fetal growth patterns are presented in Table 2. No significant differences were observed in LAZ, WAZ, or WLZ among the different patterns at 3, 6, 9, 12, 18, and 24 months of age. The changes in the WLZ showed a positive trend in the ED and SG patterns, a negative trend in the LA and EALD-H patterns, and no obvious change in the EALD-M pattern (eFigure 4 in Supplement 1, Table 2). The fetal growth patterns based on the AC Z-score were almost identical patterns (eFigure 5 in Supplement 1).

**Table 2.** Anthropometric parameters of infants aged birth to 24 months in trajectory-based subgroups.

Variables	ED (*n* = 403)	SG (*n* = 2353)	LA (*n* = 1256)	EALD-M (*n* = 1573)	EALD-H (*n* = 785)	*P*
Size at birth						
LAZ (SD)	0.01(0.84)	0.51(0.67)^a^	0.98(0.71)^ab^	0.82(0.66)^abc^	1.34(0.79)^abcd^	<0.001
WAZ (SD)	−0.60(0.75)	0.04(0.71)^a^	0.89(0.68)^ab^	0.54(0.68)^abc^	1.34(0.72)^abcd^	<0.001
WLZ (SD)	−0.70(0.74)	−0.11(0.74)^a^	0.76(0.76)^ab^	0.38(0.73)^abc^	1.21(0.81)^abcd^	<0.001
Age 3 months						
LAZ (SD)	0.63(1.13)	0.73(1.10)	0.73(1.10)	0.68(1.10)	0.72(1.16)	0.516
WAZ (SD)	0.49(0.99)	0.57(0.94)	0.62(0.95)	0.57(0.95)	0.59(0.97)	0.351
WLZ (SD)	0.17(0.89)	0.18(0.94)	0.24(0.90)	0.21(0.96)	0.22(0.90)	0.524
Change in WLZ from birth (SD)	0.87(0.07)	0.31(0.03)^a^	−0.51(0.04)^ab^	−0.20(0.04)^abc^	−1.01(0.05) ^abcd^	<0.001
Age 6 months						
LAZ (SD)	0.66(1.14)	0.74(1.12)	0.72(1.14)	0.66(1.12)	0.69(1.22)	0.202
WAZ (SD)	0.50(1.01)	0.59(0.97)	0.56(0.98)	0.59(1.00)	0.56(0.99)	0.491
WLZ (SD)	0.27(0.94)	0.31(0.95)	0.30(0.89)	0.38(0.96)	0.33(0.92)	0.140
Change in WLZ from birth (SD)	1.03(0.07)	0.46(0.03)^a^	−0.43(0.04)^ab^	0.02(0.04)^abc^	−0.89(0.05)^abcd^	<0.001
Age 9 months						
LAZ (SD)	0.58(1.10)	0.69(1.08)	0.69(1.09)	0.63(1.10)	0.74(1.16)	0.243
WAZ (SD)	0.38(1.01)	0.57(0.94)	0.57(0.97)	0.58(0.96)	0.58(0.97)	0.065
WLZ (SD)	0.22(0.98)	0.39(0.92)	0.39(0.93)	0.43(0.92)^a^	0.37(0.91)	0.028
Change in WLZ from birth (SD)	0.98(0.08)	0.50(0.03)^a^	−0.36(0.04)^ab^	0.07(0.04)^abc^	−0.84(0.05)^abcd^	<0.001
Age 12 months						
LAZ (SD)	0.56(1.14)	0.65(1.09)	0.65(1.09)	0.62(1.07)	0.64(1.17)	0.667
WAZ (SD)	0.41(0.98)	0.57(0.92)	0.55(0.93)	0.56(0.92)	0.56(0.96)	0.083
WLZ (SD)	0.24(0.94)	0.39(0.90)	0.38(0.90)	0.41(0.90)	0.39(0.92)	0.061
Change in WLZ from birth (SD)	0.99(0.08)	0.52(0.03)^a^	−0.37 (0.04)^ab^	0.04(0.04)^abc^	−0.79(0.05)^abcd^	<0.001
Age 18 months						
LAZ (SD)	0.50(1.03)	0.59(1.01)	0.59(0.97)	0.61(1.02)	0.58(1.10)	0.555
WAZ (SD)	0.40(0.92)	0.49(0.90)	0.50(0.86)	0.48(0.90)	0.46(0.92)	0.563
WLZ (SD)	0.22(0.89)	0.27(0.92)	0.30(0.87)	0.25(0.88)	0.25(0.90)	0.647
Change in WLZ from birth (SD)	0.89(0.07)	0.41(0.03)^a^	−0.37(0.04)^ab^	−0.09(0.04)^abc^	−0.94(0.05)^abcd^	<0.001
Age 24 months						
LAZ (SD)	0.58(1.06)	0.65(0.98)	0.67(1.00)	0.65(0.95)	0.71(1.05)	0.373
WAZ (SD)	0.36(0.95)	0.44(0.88)	0.46(0.86)	0.43(0.90)	0.46(0.96)	0.396
WLZ (SD)	0.03(0.86)	0.10(0.89)	0.12(0.86)	0.10(0.93)	0.11(0.93)	0.599
Change in WLZ from birth (SD)	0.66(0.07)	0.18(0.03)^a^	−0.71(0.04)^ab^	−0.32(0.04)^abc^	−1.09(0.05)^abcd^	<0.001

Trajectory subgroups: ED: early deceleration growth; SG: stable growth; LA: late acceleration growth; EALD-M: early acceleration with late decline and moderate final Z-score growth; EALD-H: early acceleration with late decline and high final Z-score growth. LAZ: length-for-age Z-score; WAZ: weight-for-age Z-score; WLZ: weight-for-length Z-score.

a, compared with the ED phenotype.

b, compared with the SG phenotype.

c, compared with the LA phenotype.

d, compared with the EALD-M phenotype.

The ED pattern exhibited the highest proportion of rapid growth at 3, 6, 9, 12, 18, and 24 months, followed by the SG pattern. The EALD-H pattern showed the highest proportion of slow growth at 3, 6, 9, 12, 18, and 24 months, followed by the LA pattern (Figure 3). The EALD-M pattern showed the highest proportion of reference range growth at 3, 9, 12, 18, and 24 months.

**Figure 3.**
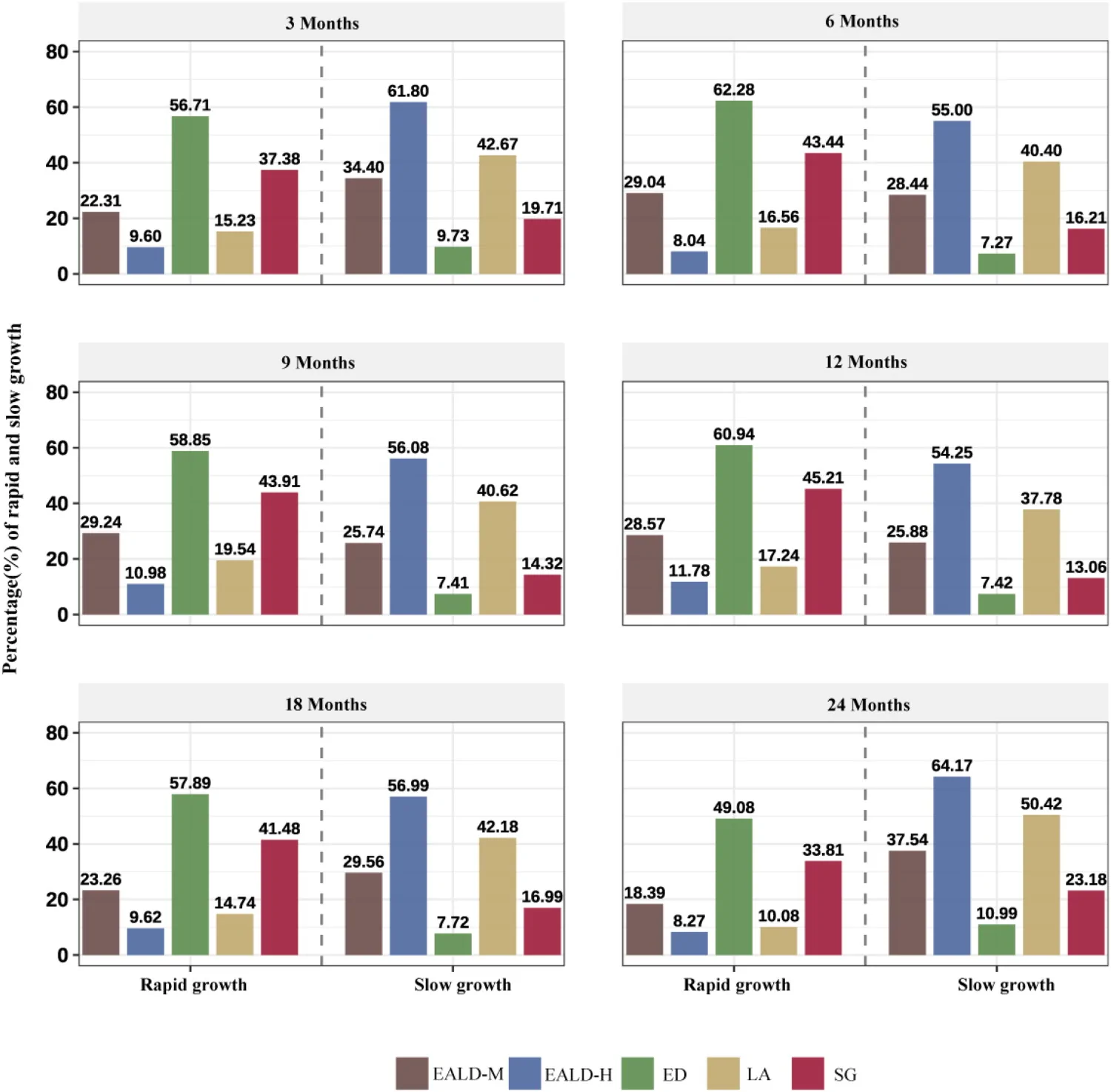
Proportions of rapid and slow growth from birth to 24 months in trajectory-based subgroups. Trajectory subgroups: EALD-M, Early acceleration with late decline and moderate final Z-score growth; EALD-H: early acceleration with late decline and high final Z-score growth; ED: early deceleration growth; LA: late acceleration growth; SG: stable growth. (A) 3 months; (B) 6 months; (C) 9 months; (D) 12 months; (E) 18 months; (F) 24 months. The sum of the rapid growth group, slow growth group and reference range group equals 100%, with the reference range group not shown here.

In the modified Poisson GEE (with EALD-M as the reference group), the results showed that ED and SG were positively associated with a higher risk of rapid growth at all time points (Figure 4(A and B)). Conversely, LA and EALD-H were associated with an increased risk of slow growth (Figure 4(C and D)).

**Figure 4.**
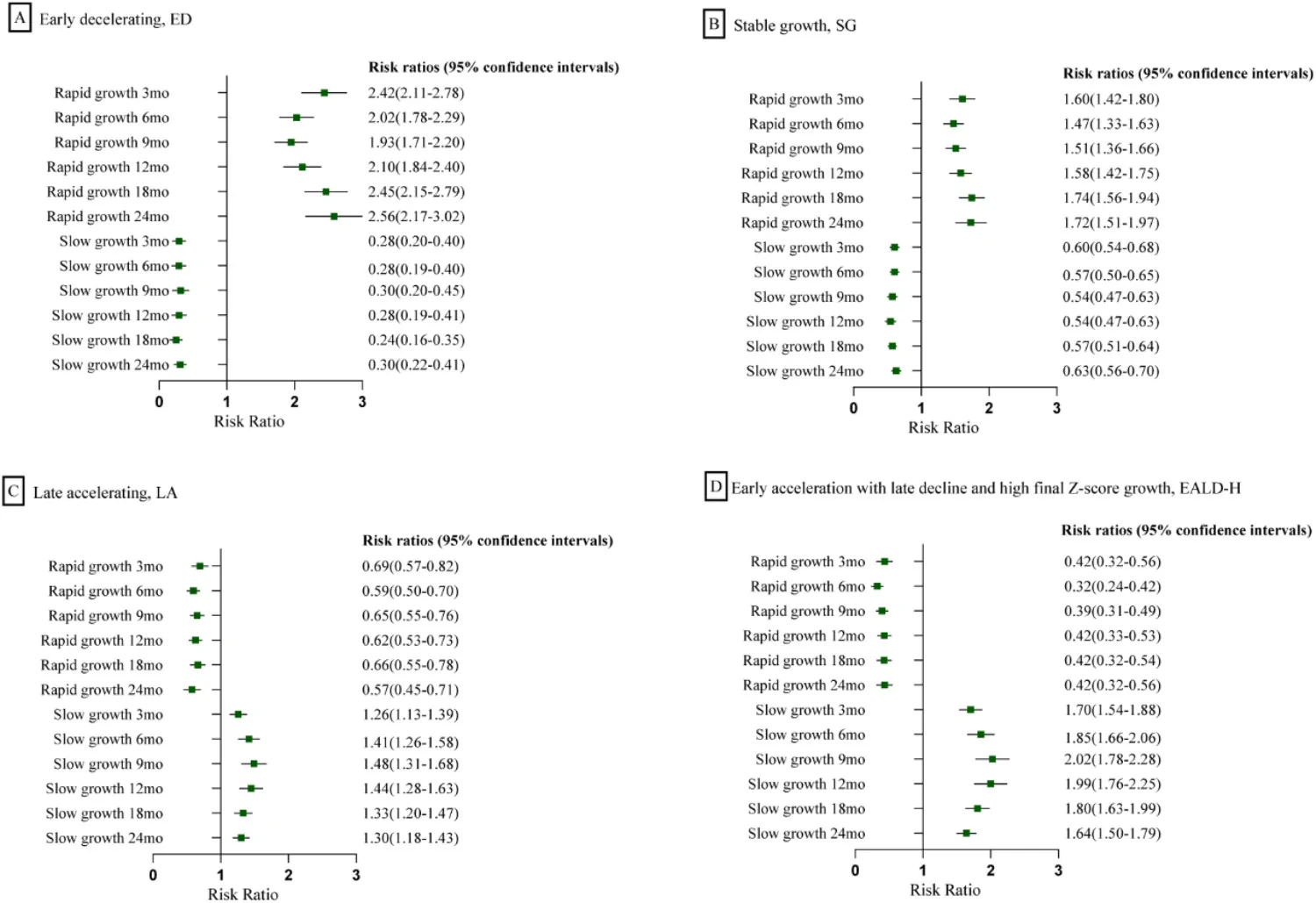
Associations between fetal growth patterns and infant growth velocity at Ages 3, 6, 9, 12, 18, and 24 Months. All models were adjusted for maternal age, prepregnancy body mass index (BMI), parity, diabetes during pregnancy, hypertensive disorders during pregnancy, maternal thyroid function and gestational weight gain.

The results of the stratified analyses are shown in eFigure 6 in Supplement 1, and the numeric results are provided in eTables 7A–D in Supplement 1. The associations for fetal growth patterns and being at risk of rapid growth in early childhood showed no statistically significant differences (all *P* for interaction ≥0.625) (eTable 8 in Supplement 1).

## Discussion

This large cohort study characterised five growth patterns; ED, SG, LA, EALD-M, and EALD-H. This classification enables a more accurate stratification of fetal growth potential. The correlation between imaging-based clustering and clinical characteristics was established by comparing clinical features across different clusters. Foetuses with the ED or SG pattern had an increased risk of rapid growth at 0–2 years of age, whereas those with the LA or EALD-H pattern had an increased risk of slow growth. These findings indicate that clustered growth patterns can serve as independent predictors of infant growth velocity and assist in identifying populations at high risk of being overweight and obese.

Traditional use of birth weight as a summary measure of fetal growth has inherent limitations [30,31]. Previous studies have confirmed that distinct growth patterns facilitate the differentiation between physiological and pathological fetal growth and exhibit significant variations in demographic characteristics, pregnancy features, and perinatal outcomes [18,19,32,33]. The risk of adverse fetal outcomes is not uniformly distributed and risk stratification based on growth patterns allows for greater precision. In this study, foetuses with different growth phenotypes had divergent perinatal characteristics, suggesting that the ED and EALD-H phenotypes may be associated with high-risk clinical profiles, potentially aiding in the identification of high-risk foetuses and offering preliminary evidence to inform clinical surveillance and personalised management of key populations. The EALD-M and EALD-H groups exhibited a reversal in growth direction after 25 weeks, with the EALD-H group showing greater deceleration than the LA group.

Previous studies on the association between fetal size and childhood growth have yielded inconsistent results [34,35], which may be due to the use of only a single time-point value without incorporating longitudinal growth data. Carolina et al. [36], based on a longitudinal cohort study including 1.7 million children, found that rapid growth from 0–2 years of age was significantly associated with a higher BMI trajectory between three and nine years of age. Giles et al. [37] reported that high growth and accelerated growth trajectories in early childhood significantly increased the risk of being overweight or obese at nine years of age. Ong et al. [38] confirmed that rapid weight gain during infancy is associated with insulin resistance. Therefore, early identification of potential growth deviations is helpful for the early detection of individuals at a high risk of being overweight or obese. Infant growth is a continuation of fetal growth, and only a few studies have explored this continuity of fetal growth [39]. In this study, infants with different fetal growth patterns showed distinct growth velocities from 0–2 years of age, suggesting that fetal growth patterns may act as early indicators for predicting infant growth velocity in the first two years of life. Therefore, fetal growth patterns may provide evidence for early intervention in obesity, thereby influencing the development of chronic diseases in adulthood.

A key observation was that 20–24 weeks of gestation may serve as an inflection point for substantial shifts in fetal growth velocity and a potentially sensitive window for modifying fetal growth trajectories, similar to the findings reported by Villar et al. [40]. He et al. [41] reported that the fetal growth rate at 22–27 weeks of gestation showed the strongest association with the BMI Z-score, LAZ, and WAZ in children at three years of age. Cao et al. [42] suggested that 22–32 weeks of gestation may represent a sensitive window for alterations in fetal growth velocity. Collectively, these observational findings imply that the second trimester could be a fetal period relevant to subsequent variations in childhood growth and development, which aligns with our study results.

This study had several limitations. First, the study included preterm infants, which may have confounded our results. However, sensitivity analyses excluding preterm births (*N* = 311) yielded growth patterns consistent with the overall analysis (eFigure 7 and eTable 9 in Supplement 1). Nevertheless, residual confounding owing to differences in gestational age at delivery across trajectory groups cannot be ruled out, warranting further validation in larger preterm cohorts or stratified studies. Second, heterogeneity may exist within the trajectory groups, and the applicability and stability of the trajectory model must be validated in a broader population. Third, this study lacked fetal growth data before 20 weeks of gestation, making it challenging to refine early pregnancy growth patterns. Incorporating placental function, such as uterine artery Doppler, will improve our understanding of the pathological mechanisms underlying trajectory classification. Future studies are warranted. Fourth, this study mainly focused on growth velocity in infants aged 0–2 years and did not explore the long-term effects of early growth velocity on outcomes, including adolescence and adulthood. Finally, this study lacked information on infant feeding owing to high missingness (>65%), leading to unmeasured confounding factors.

## Conclusion

Through multiple longitudinal measurements of intrauterine growth, our study identified five fetal growth phenotypes that better reflect the heterogeneity of fetal growth. Foetuses with ED and SG patterns have an increased risk of rapid growth from 0–2 years of age, suggesting that they may be at a high risk of rapid growth in early childhood. Gestational weeks 20–24 may serve as a sensitive window for optimising fetal growth patterns, providing new insights for the upstream prevention of childhood obesity.

## Supplementary Material

supplement1.pdf

## Data Availability

The data that support the findings of this study are available from the corresponding author, Qingqing Wu, upon reasonable request.
