# Supplementary material for: Associations of fetal growth patterns and growth in the first two years of life: a longitudinal study of a Beijing birth cohort
Source: Ann Med. 2026 Nov 2;58(1):2757376. doi: 10.1080/07853890.2026.2757376 (PMC13729623; doi:10.1080/07853890.2026.2757376)
Supplement: Supplementary file 1 [file IANN_A_2757376_SM7296.pdf]

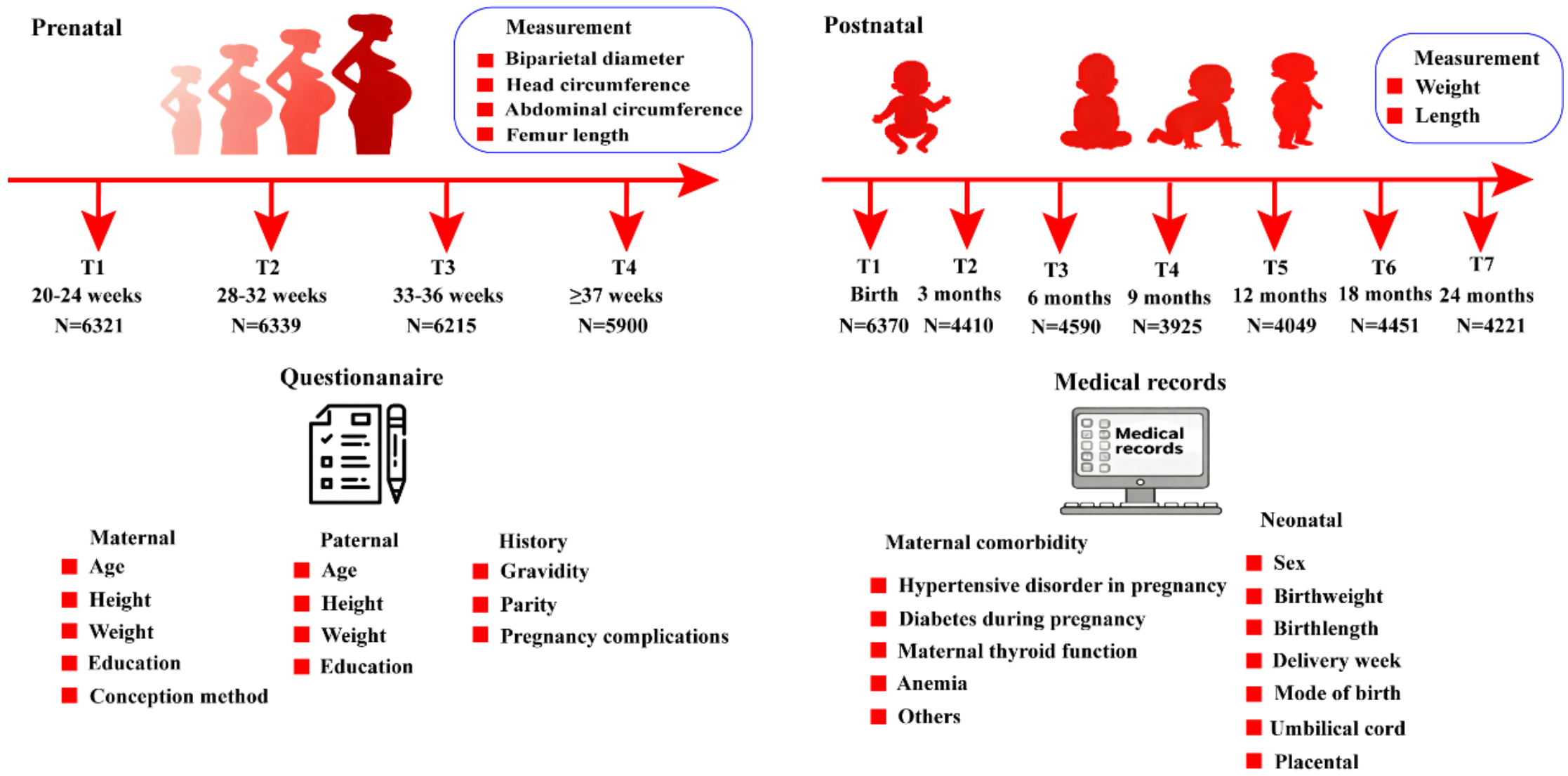

eFigure1. Research flowchart.

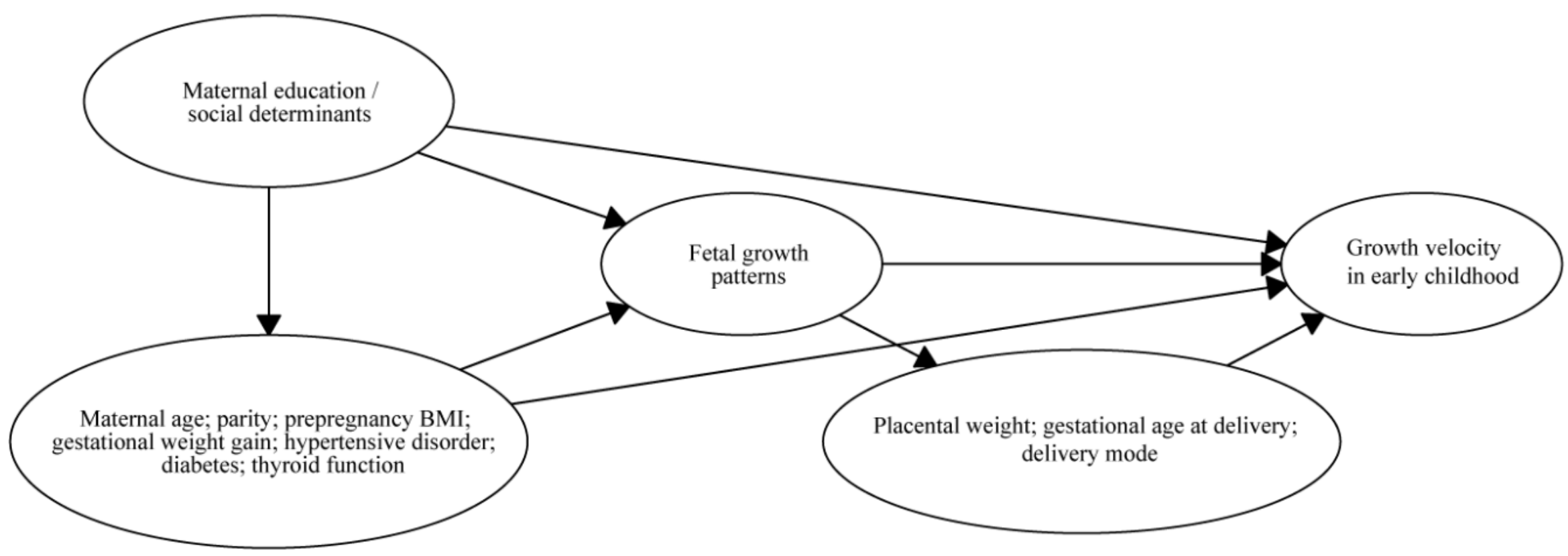

eFigure2. Research flowchart. Directed acyclic graph of the association between fetal growth patterns and growth velocity in early childhood.

eTable1. Missingness of variables

Variables	Missing data	
	n	%
Maternal factors		
Age	0	0
Educational level	0	0
Pre-pregnancy BMI	5	0.1
Parity	0	0
Diabetes during pregnancy	6	0.1
Hypertensive disorders in pregnancy	0	0
Abnormal maternal thyroid function	0	0
Gestational weight gain	6	0.1
Gestational age at delivery	0	0
Delivery mode	0	0
Maternal education	0	0
Placental weight	31	0.5
Child sex	0	0

BMI, body mass index.

eTable 2. Characteristics of parents and infants.

Variables	*n(%)*
Maternal Age (SD), y	32.9 (4.0)
Paternal Age (SD), y	32.9 (4.8)
Maternal Nation	
Han (%)	6097(95.7)
Other Nation (%)	273(4.3)
Maternal prepregnancy BMI (SD), kg/m^2	21.9 (3.3)
Maternal prepregnancy BMI Category	
18.5-24.9 (%)	4631(72.7)
<18.5 (%)	769(12.1)
25-29.9 (%)	809(12.7)
≥30 (%)	161(2.5)
Paternal BMI (SD), kg/m^2	24.8±3.3
Paternal BMI Category	
18.5-24.9 (%)	3525(55.3)
<18.5 (%)	116(1.8)
25-29.9 (%)	2305(36.2)
≥30 (%)	424(6.7)
Maternal Education	
Junior high school or below (%)	307(4.8)
Bachelor's degree or Junior college (%)	4387(68.9)
Master's degree or above (%)	1676(26.3)
Paternal Education	
Junior high school or below (%)	392(6.2)
Bachelor's degree or Junior college (%)	4297(67.4)
Master's degree or above (%)	1681(26.4)
GWG (SD), Kg	13.0±4.5
GWG Category	
Recommended (%)	2822(44.3)
Excessive (%)	1523(23.9)
Inadequate (%)	2025(31.8)
Conception	
ART (%)	366(5.8)
Natural (%)	6004(94.3)
Uniparous	
No (%)	1948(30.6)
Yes (%)	4422(69.4)
Parity	
0 time (%)	4422(69.4)
1 time (%)	1868(29.3)
≥2 times (%)	80(1.3)
Delivery week (SD), wk	38.9 (1.5)
Delivery mode	
Cesarean section (%)	2256(35.4)
Vaginal (%)	4114(64.6)
Infant sex	
Male (%)	3347 (52.5)
Female (%)	3023 (47.5)
Birthweight (SD), Kg	3.35 (0.45)

Abbreviations: BMI, body mass index (calculated as weight in kilograms divided by height in meters squared); GWG, gestational weight gain (calculated as the difference between the weight measured before childbirth and self-reported prepregnancy weight); ART, Assisted Reproductive Technology

eTable 3. Evaluation indicators of model fit for group-based trajectory modeling 1.

Group	AvepP(%)	Pj(%)	n[Pj(%)]	πj(%)	OCC	Entropy
1Groups(3)	100.0	100.0	6370(100.0)	100.0		
2Groups(33)	93.2-93.4	51.2-48.8	3261(51.2)-3109(48.8)	50.9-49.1	13.28-14.80	0.775
3Groups(333)	90.0-90.2-90.5	24.8-56.4-18.8	1581(24.8)-3590(56.4)-1199(18.8)	25.3-55.1-19.6	26.52-7.47-38.89	0.790
4Groups(3333)	88.5-88.3-87.8-89.5	6.4-38.6-43.4-11.5	410(6.4)-2462(38.6)-2764(43.4)-734(11.5)	6.9-38.5-42.6-11.9	104.07-12.01-9.66-62.79	0.798
5Groups(33333)	89.4-87.2-75.2-77.7-88.7	6.3-36.9-19.7-24.7-12.3	403(6.3)-2353(36.9)-1256(19.7)-1573(24.7)-785(12.3)	6.7-35.8-20.8-24.4-12.2	117.09-12.22-11.56-10.79-56.51	0.742
6Groups(333333)	79.8-77.7-78.5-79.5-89.4-87.3	14.3-26.7-18.3-25.4-9.9-5.3	910(14.3)-1703(26.7)-1166(18.3)-1620(25.4)-631(9.9)-340(5.3)	15.0-26.1-18.8-24.9-9.9-5.3	22.43-9.91-15.78-11.68-76.07-122.03	0.731

Note: Good model fit indicated by (1)Avepp(Average posterior probability)greater than 0.7 for each group,(2)Pj(Posterior probability of group membership) greater than 5%,(3)Close correspondencePj and πj(Probability of group membership),(4) BIC(Bayesian information criterion) close to 0,(5)The large △BIC(BIC difference between complex and simple models) the more complex models are accepted,(6)OCC(Odds of Correct Classification)greater than 5,(7)E(Relative entropy) greater than 0.7.

eTable 4. Evaluation indicators of model fit for group-based trajectory modeling 2.

Group	Parameters	LogLik	BIC^1	ΔBIC^1	BIC^2	ΔBIC^2	AIC	LRT
1Groups(3)	8	-37806.4	-37828.3	—	-37832.3	—	-37811.4	—
2Groups(33)	12	-33370.3	-33414.1	4414.200	-33422.1	4410.0	-33380.3	<0.001
3Groups(333)	16	-31809.9	-31875.6	1538.500	-31887.5	1535.0	-31824.9	<0.001
4Groups(3333)	20	-31098.1	-31185.7	689.900	-31201.5	686.0	-31118.1	<0.001
5Groups(33333)	24	-30736.6	-30846.1	339.600	-30865.9	336.0	-30761.6	<0.001
6Groups(333333)	28	-30330.0	-30461.4	384.700	-30485.2	381.0	-30360.0	<0.001

Model Selection

The results showed that the 1-group model failed to characterize the heterogeneity of fetal growth trajectories. The relative entropies of the 2-group and 3-group models were 0.775 and 0.790, respectively. Although they met the fitting requirements, the trajectory classification was too coarse to fully reveal the growth differences within the fetal population. The 4-group model contained a subgroup accounting for only 6.4% of the total sample, with a small sample size and insufficiently refined trajectory characterization. The 6-group model had a minor group representing only 5.3%, close to the 5% threshold; meanwhile, the average posterior probabilities of some groups were low, indicating overfitting and redundant interpretation. In contrast, all fitting indices of the 5-group model reached excellent standards. The average posterior probabilities of each group were 89.4%, 87.2%, 75.2%, 77.4%, and 88.7%, all above 0.7. The proportion of each group exceeded 5%, and Pj was highly consistent with πj, demonstrating good classification reliability. The OCC of all groups were greater than 5, indicating high classification accuracy. The relative entropy was 0.742, higher than 0.7, suggesting that the model had stable and reliable discrimination ability for different trajectory groups and could effectively characterize the heterogeneity of fetal growth trajectories.

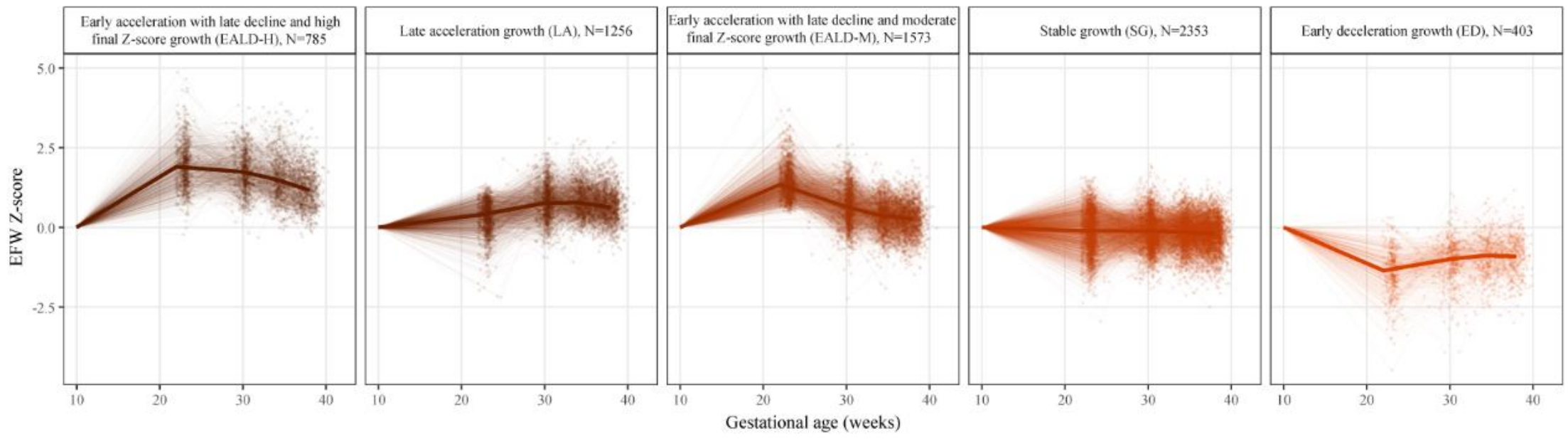

eFigure3. Individual EFW growth trajectories. Consistent with the original trajectory specification, each thin within-child line begins at the common anchor of 10 gestational weeks and a z score of 0; points show observed measurements at subsequent visits. The thick line and shaded band reproduce the fitted trajectory and 95% confidence interval from the original model; no trajectory was refitted or reassigned.

eTable5. Comparison between cluster membership and classification by birth weight and gestational age. Values are given as n (column %)

	AGA	SGA	LGA
Early decelerating (ED)	327(6.4%)	73(46.5%)	3(0.2%)
Stable growth (SG)	2182(42.4%)	77(49.0%)	94(8.8%)
Late accelerating (LA)	892(17.4%)	0	364(34.0%)
Early accelerating with late decelerating and moderate final Z-score growth (EALD-M)	1358(26.4%)	7(4.5%)	208(19.4%)
Early accelerating with ate decline and high final Z-score growth (EALD-H)	382(7.4%)	0	403(37.6%)

Abbreviations: AGA, Appropriate-for-gestational-age; LGA, Large-for-gestational-age; SGA, Small-for-gestational-age

eTable6. Delivery and neonatal characteristics in trajectory-based subgroups.

Variables	ED (n=403)	SG (n=2353)	LA (n=1256)	EALD-M (n=1573)	EALD-H (n=785)	P
Delivery time	38.79±2.02	39.03±1.40	38.95±1.42	38.76±1.45	38.67±1.32	<0.001
Delivery Mode						<0.001
Cesarean section	158(39.21)	692(29.41)	504(40.13)	529(33.63)	373(47.52)	
Vaginal delivery	245(60.79)	1661(70.59)	752(59.87)	1044(66.37)	412(52.48)	
Preterm Birth						<0.001
No	366 (90.82)	2257 (95.92)	1201 (95.62)	1490 (94.72)	745 (94.90)	
Yes	37 (9.18)	96 (4.08)	55 (4.38)	83 (5.28)	40 (5.10)	
Infant sex						<0.001
Male	140 (34.74)	1069 (45.43)	639 (50.88)	963 (61.26)	535 (68.15)	
Female	263 (65.26)	1284 (54.57)	617 (49.12)	609 (38.74)	250 (31.85)	
Birth Weight	2874.4±488.6	3188.3±379.6a	3539.3±396.9ab	3368.0±376.8abc	3724.6±421.4abcd	<0.001
Placental Surface Area	265.1±44.9	281.5±39.0a	296.5±46.4ab	287.5±42.3abc	306.3±50.5abcd	<0.001
Placental Volume	375.9±93.2	406.8±100.1a	454.3±136.1ab	423.3±112.4abc	476.5±145.3abcd	<0.001
Placental Weight	544.6±94.3	576.9±90.4a	631.7±123.9ab	594.3±96.2abc	668.1±140.8abcd	<0.001
BW/PW	5.35±0.89	5.61±0.83a	5.75±0.97ab	5.76±0.85ab	5.73±0.97ab	<0.001
Umbilical cord length	52.4±9.4	54.7±8.1a	55.7±8.2ab	55.6±8.2ab	56.6±8.9abd	<0.001
Torsion of cord						0.003
No	380 (94.29)	2277 (96.77)	1229 (97.85)	1534 (97.52)	766 (97.58)	
Yes	23 (5.71)	76 (3.23)	27 (2.15)	39 (2.48)	19 (2.42)	
Umbilical cord around neck						0.825
No	301 (74.69)	1713 (72.80)	934 (74.36)	1148 (72.98)	577 (73.50)	
Yes	102 (25.31)	640 (27.20)	322 (25.64)	425 (27.02)	208 (26.50)	
Cord Insertion						0.066
Normal	376 (93.30)	2254 (95.79)	1214 (96.66)	1509 (95.93)	754 (96.05)	
Abnormal	27 (6.70)	99 (4.21)	42 (3.34)	64 (4.07)	31 (3.95)	

Abbreviations: ED, Early deceleration growth; SG, Stable growth; LA, Late acceleration growth; EALD-M, early acceleration with late deceleration and moderate final Z-score growth; EALD-H, early acceleration with late decline and high final Z-score growth.
a, compared with the ED phenotype;
b, compared with the SG phenotype;
c, compared with the LA phenotype;
d, compared with the EALD phenotype.

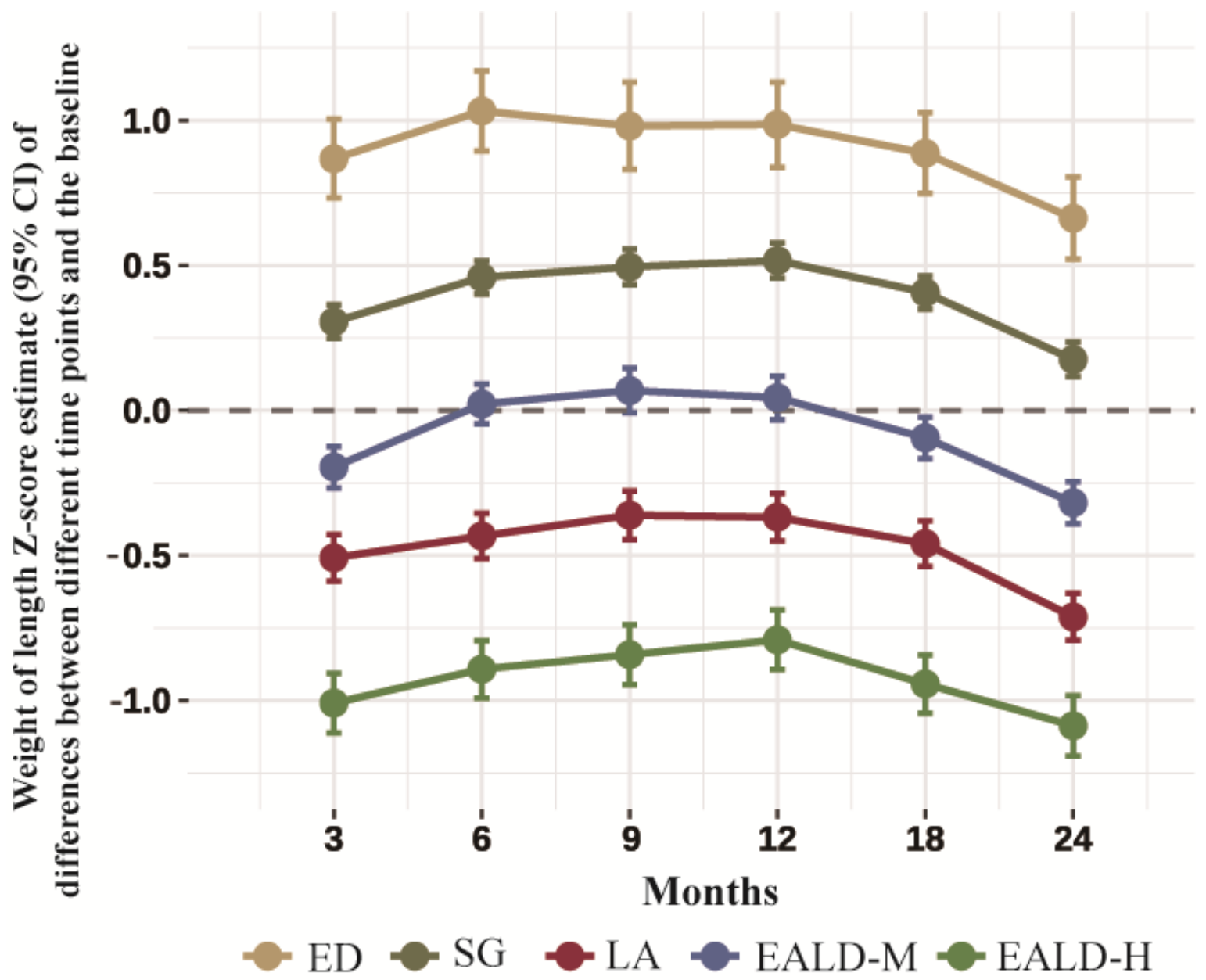

eFigure 4. The trend of the effect of changes in weight-for-length Z-score from birth to 24 months of age among infants in different growth trajectory subgroups based on EFW Z-score. ED, Early deceleration growth; SG, Stable growth; LA, Late acceleration growth; EALD-M, early acceleration with late deceleration and moderate final Z-score growth; EALD-H, early acceleration with late decline and high final Z-score growth.

Abbreviations: EFW, estimated fetal weight.

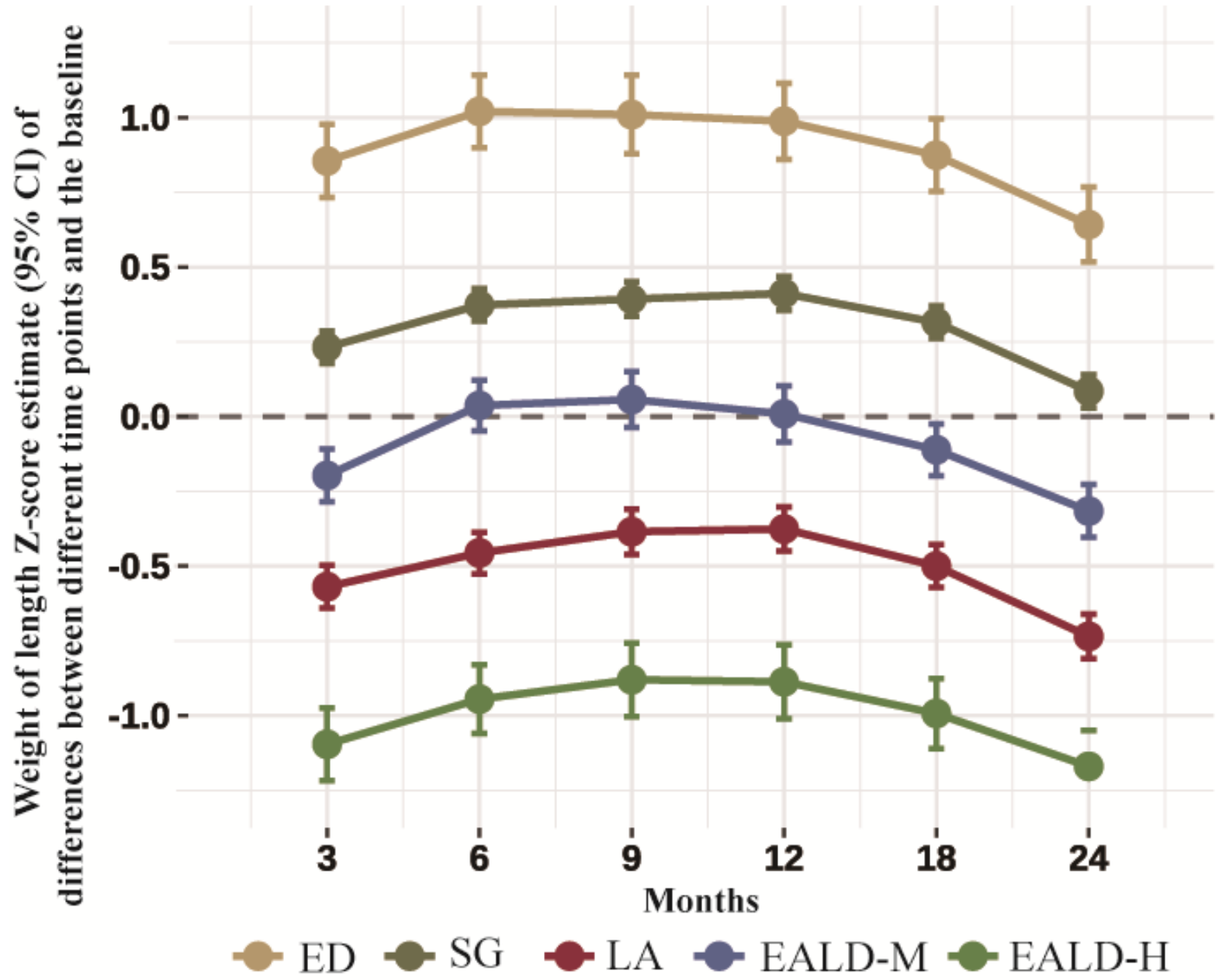

eFigure 5. The trend of the effect of changes in weight-for-length Z-score from birth to 24 months of age among infants in different growth trajectory subgroups based on AC Z-score. ED, Early deceleration growth; SG, Stable growth; LA, Late acceleration growth; EALD-M, early acceleration with late deceleration and moderate final Z-score growth; EALD-H, early acceleration with late decline and high final Z-score growth

Abbreviations: AC, abdominal circumstance.

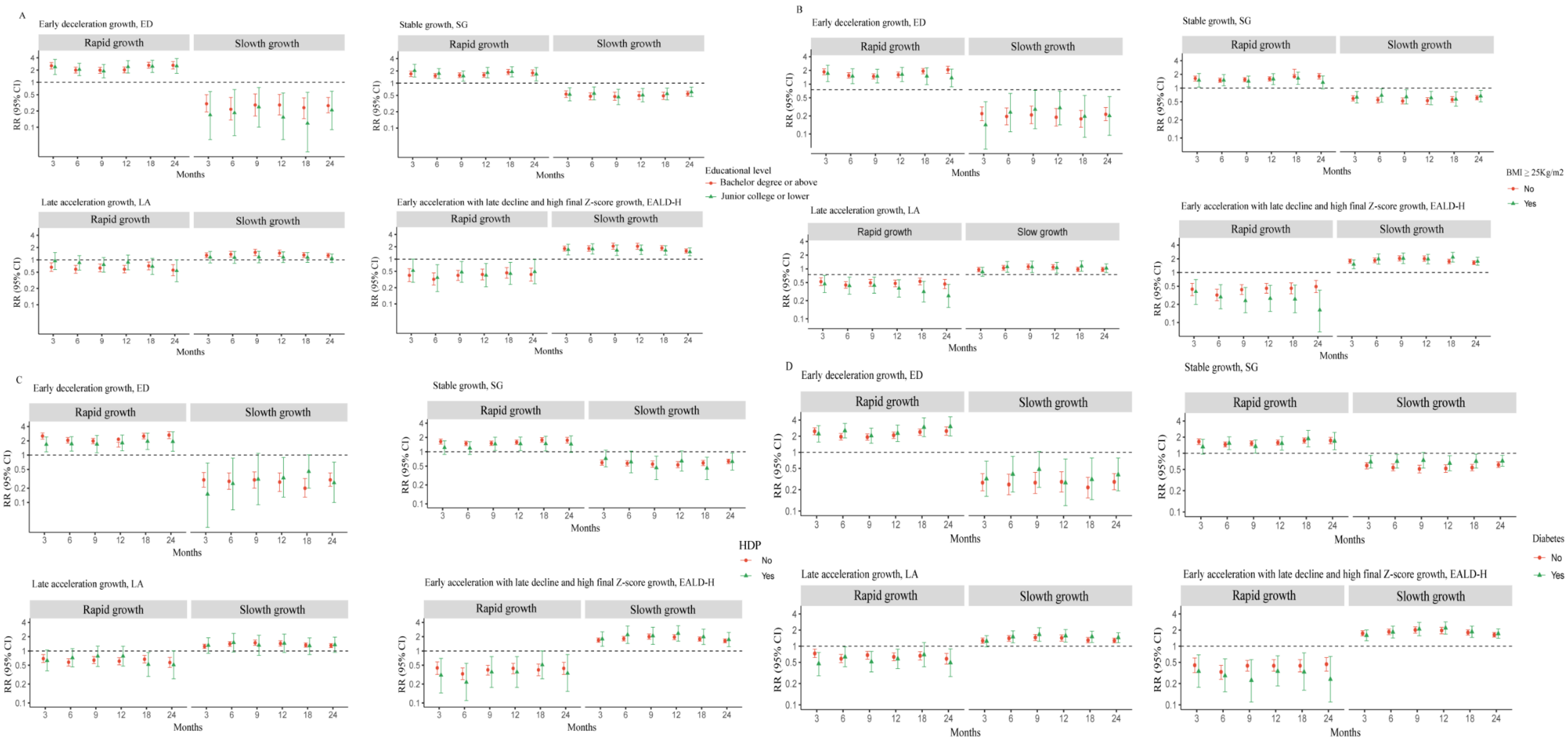

eFigure 6. Analyses stratified by maternal educational level (A), prepregnancy body mass index (BMI) (B), hypertensive disorder in pregnancy (HDP)(C) and diabetes during pregnancy (D) for associations between fetal growth patterns and growth velocity in early childhood.

eTable 7A. Associations between fetal growth patterns and growth velocity in early childhood stratified by maternal education level

Stratum	Growth velocity	Comparison	3 months	6 months	9 months	12 months	18 months	24 months
Bachelor degree or above	Rapid	ED vs EALD-M	2.55 (2.12–3.06)	2.00 (1.69–2.37)	1.98 (1.67–2.34)	2.02 (1.71–2.39)	2.62 (2.22–3.10)	2.62 (2.12–3.23)
Bachelor degree or above	Rapid	SG vs EALD-M	1.69 (1.45–1.98)	1.52 (1.34–1.73)	1.56 (1.37–1.79)	1.58 (1.39–1.81)	1.86 (1.61–2.16)	1.78 (1.50–2.12)
Bachelor degree or above	Rapid	LA vs EALD-M	0.66 (0.52–0.85)	0.59 (0.47–0.73)	0.63 (0.51–0.79)	0.59 (0.48–0.73)	0.71 (0.57–0.88)	0.56 (0.41–0.76)
Bachelor degree or above	Rapid	EALD-H vs EALD-M	0.40 (0.28–0.58)	0.32 (0.23–0.46)	0.40 (0.30–0.53)	0.42 (0.31–0.57)	0.46 (0.34–0.62)	0.42 (0.29–0.60)
Bachelor degree or above	Slow	ED vs EALD-M	0.30 (0.19–0.50)	0.22 (0.12–0.43)	0.28 (0.15–0.52)	0.28 (0.16–0.51)	0.24 (0.13–0.42)	0.27 (0.18–0.43)
Bachelor degree or above	Slow	SG vs EALD-M	0.54 (0.45–0.65)	0.48 (0.39–0.58)	0.47 (0.38–0.60)	0.50 (0.40–0.62)	0.49 (0.40–0.60)	0.55 (0.48–0.64)
Bachelor degree or above	Slow	LA vs EALD-M	1.29 (1.12–1.49)	1.37 (1.17–1.61)	1.52 (1.27–1.82)	1.46 (1.22–1.73)	1.31 (1.13–1.52)	1.28 (1.13–1.45)
Bachelor degree or above	Slow	EALD-H vs EALD-M	1.80 (1.57–2.07)	1.81 (1.54–2.12)	2.07 (1.75–2.46)	2.05 (1.73–2.44)	1.86 (1.62–2.15)	1.58 (1.40–1.78)
Junior college or lower	Rapid	ED vs EALD-M	2.39 (1.56–3.67)	2.07 (1.46–2.95)	1.88 (1.29–2.75)	2.40 (1.69–3.40)	2.47 (1.75–3.48)	2.53 (1.67–3.83)
Junior college or lower	Rapid	SG vs EALD-M	2.02 (1.41–2.89)	1.72 (1.30–2.28)	1.50 (1.12–1.99)	1.81 (1.35–2.43)	1.90 (1.42–2.54)	1.66 (1.14–2.41)
Junior college or lower	Rapid	LA vs EALD-M	0.94 (0.58–1.52)	0.85 (0.56–1.27)	0.76 (0.50–1.14)	0.87 (0.57–1.31)	0.69 (0.44–1.09)	0.54 (0.29–1.00)
Junior college or lower	Rapid	EALD-H vs EALD-M	0.52 (0.27–1.00)	0.35 (0.16–0.73)	0.48 (0.27–0.87)	0.40 (0.21–0.78)	0.44 (0.24–0.83)	0.49 (0.25–0.98)
Junior college or lower	Slow	ED vs EALD-M	0.16 (0.04–0.60)	0.18 (0.05–0.67)	0.25 (0.08–0.75)	0.14 (0.04–0.55)	0.10 (0.02–0.57)	0.21 (0.07–0.61)
Junior college or lower	Slow	SG vs EALD-M	0.53 (0.37–0.77)	0.56 (0.39–0.81)	0.46 (0.30–0.71)	0.51 (0.35–0.74)	0.55 (0.39–0.76)	0.61 (0.47–0.80)
Junior college or lower	Slow	LA vs EALD-M	1.16 (0.84–1.60)	1.14 (0.82–1.60)	1.17 (0.85–1.61)	1.16 (0.86–1.57)	1.15 (0.87–1.52)	1.09 (0.86–1.38)
Junior college or lower	Slow	EALD-H vs EALD-M	1.70 (1.26–2.29)	1.77 (1.32–2.37)	1.64 (1.22–2.21)	1.69 (1.29–2.21)	1.63 (1.25–2.13)	1.50 (1.21–1.87)

Trajectory groups: ED, Early deceleration growth; SG, Stable growth; LA, Late acceleration growth; EALD-M, Early acceleration with late decline and moderate final Z-score growth; EALD-H, Early acceleration with late decline and high final Z-score growth.

eTable 7B. Associations between fetal growth patterns and growth velocity in early childhood stratified by maternal prepregnancy body mass index (BMI)

Stratum	Growth velocity	Comparison	3 months	6 months	9 months	12 months	18 months	24 months
BMI $<$25Kg/m^2	Rapid	ED vs EALD-M	2.48 (2.14–2.88)	2.06 (1.81–2.36)	1.97 (1.72–2.25)	2.14 (1.86–2.45)	2.57 (2.24–2.94)	2.76 (2.31–3.29)
BMI $<$25Kg/m^2	Rapid	SG vs EALD-M	1.63 (1.43–1.84)	1.48 (1.33–1.65)	1.53 (1.38–1.70)	1.59 (1.43–1.78)	1.78 (1.58–2.00)	1.82 (1.58–2.09)
BMI $<$25Kg/m^2	Rapid	LA vs EALD-M	0.70 (0.57–0.85)	0.59 (0.50–0.70)	0.66 (0.55–0.78)	0.64 (0.54–0.76)	0.71 (0.59–0.84)	0.62 (0.49–0.78)
BMI $<$25Kg/m^2	Rapid	EALD-H vs EALD-M	0.43 (0.31–0.58)	0.32 (0.24–0.43)	0.42 (0.33–0.54)	0.45 (0.35–0.58)	0.45 (0.34–0.59)	0.49 (0.36–0.67)
BMI $<$25Kg/m^2	Slow	ED vs EALD-M	0.30 (0.21–0.42)	0.26 (0.17–0.40)	0.28 (0.18–0.44)	0.25 (0.16–0.39)	0.23 (0.15–0.35)	0.29 (0.21–0.41)
BMI $<$25Kg/m^2	Slow	SG vs EALD-M	0.59 (0.52–0.68)	0.55 (0.47–0.63)	0.52 (0.45–0.61)	0.53 (0.44–0.62)	0.56 (0.49–0.65)	0.61 (0.55–0.69)
BMI $<$25Kg/m^2	Slow	LA vs EALD-M	1.28 (1.15–1.44)	1.40 (1.24–1.59)	1.49 (1.30–1.71)	1.45 (1.27–1.66)	1.30 (1.16–1.45)	1.29 (1.16–1.44)
BMI $<$25Kg/m^2	Slow	EALD-H vs EALD-M	1.78 (1.59–1.98)	1.84 (1.63–2.08)	2.04 (1.79–2.34)	2.01 (1.75–2.31)	1.75 (1.56–1.96)	1.64 (1.49–1.81)
BMI $\geqslant$25Kg/m^2	Rapid	ED vs EALD-M	2.29 (1.52–3.46)	1.99 (1.37–2.89)	1.99 (1.41–2.82)	2.17 (1.52–3.11)	1.99 (1.30–3.05)	1.82 (1.16–2.84)
BMI $\geqslant$25Kg/m^2	Rapid	SG vs EALD-M	1.49 (1.05–2.10)	1.49 (1.12–1.98)	1.41 (1.07–1.84)	1.58 (1.19–2.08)	1.64 (1.21–2.24)	1.33 (0.96–1.85)
BMI $\geqslant$25Kg/m^2	Rapid	LA vs EALD-M	0.62 (0.40–0.96)	0.57 (0.37–0.88)	0.58 (0.39–0.88)	0.50 (0.32–0.77)	0.42 (0.25–0.71)	0.34 (0.19–0.61)
BMI $\geqslant$25Kg/m^2	Rapid	EALD-H vs EALD-M	0.38 (0.20–0.70)	0.29 (0.16–0.54)	0.24 (0.13–0.47)	0.27 (0.14–0.52)	0.26 (0.13–0.53)	0.15 (0.05–0.41)
BMI $\geqslant$25Kg/m^2	Slow	ED vs EALD-M	0.17 (0.05–0.55)	0.32 (0.12–0.83)	0.37 (0.14–0.97)	0.40 (0.17–0.93)	0.26 (0.09–0.75)	0.27 (0.10–0.71)
BMI $\geqslant$25Kg/m^2	Slow	SG vs EALD-M	0.63 (0.47–0.84)	0.69 (0.49–0.97)	0.64 (0.45–0.92)	0.61 (0.43–0.85)	0.57 (0.40–0.83)	0.67 (0.50–0.89)
BMI $\geqslant$25Kg/m^2	Slow	LA vs EALD-M	1.15 (0.92–1.44)	1.48 (1.12–1.94)	1.47 (1.11–1.97)	1.41 (1.08–1.86)	1.54 (1.18–2.00)	1.38 (1.11–1.72)
BMI $\geqslant$25Kg/m^2	Slow	EALD-H vs EALD-M	1.51 (1.21–1.89)	1.98 (1.53–2.55)	2.05 (1.57–2.68)	1.97 (1.55–2.50)	2.17 (1.69–2.78)	1.76 (1.44–2.16)

Trajectory groups: ED, Early deceleration growth; SG, Stable growth; LA, Late acceleration growth; EALD-M, Early acceleration with late decline and moderate final Z-score growth; EALD-H, Early acceleration with late decline and high final Z-score growth.

eTable 7C. Associations between fetal growth patterns and growth velocity in early childhood stratified by hypertensive disorder in pregnancy

Stratum	Growth velocity	Comparison	3 months	6 months	9 months	12 months	18 months	24 months
No	Rapid	ED vs EALD-M	2.52 (2.17–2.93)	2.05 (1.79–2.35)	1.98 (1.73–2.26)	2.15 (1.87–2.47)	2.51 (2.19–2.88)	2.64 (2.22–3.14)
No	Rapid	SG vs EALD-M	1.64 (1.45–1.86)	1.50 (1.35–1.67)	1.51 (1.36–1.68)	1.59 (1.43–1.77)	1.77 (1.58–1.98)	1.75 (1.52–2.01)
No	Rapid	LA vs EALD-M	0.69 (0.57–0.84)	0.58 (0.48–0.69)	0.64 (0.54–0.76)	0.61 (0.51–0.72)	0.67 (0.56–0.81)	0.57 (0.45–0.73)
No	Rapid	EALD-H vs EALD-M	0.44 (0.32–0.59)	0.33 (0.25–0.44)	0.40 (0.31–0.50)	0.43 (0.33–0.55)	0.40 (0.30–0.53)	0.43 (0.32–0.58)
No	Slow	ED vs EALD-M	0.30 (0.21–0.43)	0.28 (0.19–0.42)	0.30 (0.20–0.44)	0.27 (0.17–0.42)	0.20 (0.13–0.32)	0.30 (0.22–0.42)
No	Slow	SG vs EALD-M	0.59 (0.52–0.67)	0.57 (0.50–0.65)	0.55 (0.47–0.64)	0.53 (0.46–0.62)	0.58 (0.51–0.66)	0.63 (0.56–0.70)
No	Slow	LA vs EALD-M	1.25 (1.13–1.39)	1.41 (1.25–1.58)	1.49 (1.31–1.70)	1.44 (1.27–1.64)	1.33 (1.20–1.48)	1.30 (1.18–1.43)
No	Slow	EALD-H vs EALD-M	1.69 (1.53–1.88)	1.82 (1.63–2.04)	2.01 (1.77–2.28)	1.96 (1.72–2.22)	1.78 (1.61–1.98)	1.63 (1.49–1.79)
Yes	Rapid	ED vs EALD-M	1.68 (1.17–2.41)	1.72 (1.21–2.45)	1.70 (1.12–2.59)	1.80 (1.23–2.65)	1.95 (1.31–2.89)	1.93 (1.20–3.10)
Yes	Rapid	SG vs EALD-M	1.23 (0.88–1.70)	1.20 (0.88–1.64)	1.46 (1.06–2.03)	1.47 (1.05–2.04)	1.48 (1.04–2.12)	1.45 (0.97–2.16)
Yes	Rapid	LA vs EALD-M	0.63 (0.38–1.05)	0.72 (0.46–1.12)	0.78 (0.47–1.27)	0.78 (0.48–1.26)	0.52 (0.29–0.93)	0.51 (0.26–1.01)
Yes	Rapid	EALD-H vs EALD-M	0.31 (0.13–0.70)	0.22 (0.09–0.55)	0.36 (0.17–0.76)	0.36 (0.17–0.76)	0.51 (0.26–1.00)	0.34 (0.14–0.84)
Yes	Slow	ED vs EALD-M	0.15 (0.03–0.68)	0.25 (0.07–0.86)	0.31 (0.09–1.09)	0.33 (0.13–0.89)	0.45 (0.20–1.01)	0.26 (0.10–0.71)
Yes	Slow	SG vs EALD-M	0.72 (0.48–1.08)	0.61 (0.36–1.03)	0.46 (0.26–0.81)	0.64 (0.39–1.04)	0.45 (0.26–0.77)	0.62 (0.41–0.94)
Yes	Slow	LA vs EALD-M	1.31 (0.89–1.91)	1.50 (0.95–2.35)	1.32 (0.81–2.15)	1.45 (0.93–2.26)	1.29 (0.84–1.98)	1.35 (0.94–1.96)
Yes	Slow	EALD-H vs EALD-M	1.79 (1.26–2.54)	2.20 (1.44–3.36)	2.09 (1.38–3.16)	2.35 (1.61–3.42)	1.99 (1.37–2.89)	1.74 (1.23–2.45)

Trajectory groups: ED, Early deceleration growth; SG, Stable growth; LA, Late acceleration growth; EALD-M, Early acceleration with late decline and moderate final Z-score growth; EALD-H, Early acceleration with late decline and high final Z-score growth.

eTable 7D. Associations between fetal growth patterns and growth velocity in early childhood stratified by diabetes during pregnancy

Stratum	Growth velocity	Comparison	3 months	6 months	9 months	12 months	18 months	24 months
No	Rapid	ED vs EALD-M	2.46 (2.12–2.86)	1.95 (1.69–2.24)	1.92 (1.68–2.20)	2.08 (1.81–2.39)	2.39 (2.08–2.73)	2.49 (2.08–2.97)
No	Rapid	SG vs EALD-M	1.65 (1.46–1.87)	1.46 (1.31–1.63)	1.53 (1.38–1.70)	1.58 (1.42–1.77)	1.73 (1.54–1.94)	1.73 (1.50–1.98)
No	Rapid	LA vs EALD-M	0.73 (0.61–0.88)	0.58 (0.49–0.70)	0.68 (0.57–0.80)	0.63 (0.53–0.75)	0.66 (0.55–0.79)	0.58 (0.46–0.73)
No	Rapid	EALD-H vs EALD-M	0.44 (0.32–0.60)	0.33 (0.24–0.44)	0.43 (0.34–0.54)	0.43 (0.34–0.56)	0.43 (0.33–0.56)	0.46 (0.34–0.61)
No	Slow	ED vs EALD-M	0.27 (0.19–0.40)	0.25 (0.16–0.39)	0.27 (0.17–0.42)	0.28 (0.18–0.43)	0.22 (0.14–0.34)	0.28 (0.20–0.40)
No	Slow	SG vs EALD-M	0.59 (0.51–0.67)	0.54 (0.47–0.63)	0.51 (0.43–0.60)	0.52 (0.44–0.61)	0.54 (0.47–0.62)	0.61 (0.54–0.69)
No	Slow	LA vs EALD-M	1.26 (1.13–1.42)	1.40 (1.23–1.58)	1.45 (1.27–1.66)	1.42 (1.24–1.63)	1.29 (1.15–1.45)	1.27 (1.15–1.41)
No	Slow	EALD-H vs EALD-M	1.73 (1.55–1.94)	1.85 (1.64–2.09)	2.01 (1.75–2.30)	1.96 (1.70–2.25)	1.80 (1.61–2.01)	1.63 (1.48–1.80)
Yes	Rapid	ED vs EALD-M	2.21 (1.55–3.14)	2.53 (1.85–3.46)	2.03 (1.47–2.82)	2.26 (1.59–3.22)	2.93 (1.95–4.40)	3.03 (2.01–4.58)
Yes	Rapid	SG vs EALD-M	1.32 (0.96–1.82)	1.54 (1.17–2.03)	1.34 (1.01–1.77)	1.53 (1.14–2.06)	1.88 (1.32–2.66)	1.69 (1.16–2.46)
Yes	Rapid	LA vs EALD-M	0.47 (0.28–0.76)	0.63 (0.41–0.97)	0.51 (0.33–0.80)	0.58 (0.38–0.88)	0.69 (0.41–1.17)	0.49 (0.27–0.89)
Yes	Rapid	EALD-H vs EALD-M	0.34 (0.17–0.69)	0.28 (0.14–0.58)	0.23 (0.09–0.56)	0.34 (0.18–0.66)	0.33 (0.15–0.75)	0.24 (0.09–0.64)
Yes	Slow	ED vs EALD-M	0.32 (0.15–0.68)	0.39 (0.18–0.84)	0.48 (0.22–1.03)	0.27 (0.10–0.75)	0.31 (0.13–0.78)	0.38 (0.19–0.78)
Yes	Slow	SG vs EALD-M	0.69 (0.53–0.91)	0.71 (0.53–0.95)	0.74 (0.53–1.04)	0.65 (0.47–0.90)	0.71 (0.53–0.96)	0.72 (0.57–0.92)
Yes	Slow	LA vs EALD-M	1.24 (0.97–1.57)	1.49 (1.15–1.93)	1.65 (1.23–2.21)	1.56 (1.19–2.04)	1.50 (1.17–1.91)	1.44 (1.16–1.78)
Yes	Slow	EALD-H vs EALD-M	1.59 (1.26–2.00)	1.83 (1.42–2.37)	2.09 (1.56–2.78)	2.18 (1.68–2.84)	1.85 (1.45–2.37)	1.71 (1.40–2.10)

Trajectory groups: ED, Early deceleration growth; SG, Stable growth; LA, Late acceleration growth; EALD-M, Early acceleration with late decline and moderate final Z-score growth; EALD-H, Early acceleration with late decline and high final Z-score growth.

eTable8. Interaction tests for the associations between fetal growth patterns and growth velocity in early childhood

Modifier	Growth velocity	Interaction test	df	Statistic	P value
Maternal education level	Rapid	Trajectory-by-determinant interaction across ages	48	27.91	0.991
Maternal education level	Rapid	Trajectory-by-age-by-determinant interaction	40	21.95	0.991
Maternal education level	Slow	Trajectory-by-determinant interaction across ages	48	35.31	0.913
Maternal education level	Slow	Trajectory-by-age-by-determinant interaction	40	17.46	0.999
Maternal prepregnancy body mass index	Rapid	Trajectory-by-determinant interaction across ages	24	17.03	0.847
Maternal prepregnancy body mass index	Rapid	Trajectory-by-age-by-determinant interaction	20	13.19	0.869
Maternal prepregnancy body mass index	Slow	Trajectory-by-determinant interaction across ages	24	18.42	0.782
Maternal prepregnancy body mass index	Slow	Trajectory-by-age-by-determinant interaction	20	17.43	0.625
Hypertensive disorder in pregnancy	Rapid	Trajectory-by-determinant interaction across ages	24	17.97	0.804
Hypertensive disorder in pregnancy	Rapid	Trajectory-by-age-by-determinant interaction	20	11.84	0.921
Hypertensive disorder in pregnancy	Slow	Trajectory-by-determinant interaction across ages	24	16.14	0.883
Hypertensive disorder in pregnancy	Slow	Trajectory-by-age-by-determinant interaction	20	14.80	0.788
Diabetes during pregnancy	Rapid	Trajectory-by-determinant interaction across ages	24	15.03	0.920
Diabetes during pregnancy	Rapid	Trajectory-by-age-by-determinant interaction	20	11.14	0.942
Diabetes during pregnancy	Slow	Trajectory-by-determinant interaction across ages	24	14.37	0.938
Diabetes during pregnancy	Slow	Trajectory-by-age-by-determinant interaction	20	7.81	0.993

No interaction test was statistically significant; all P values were ≥0.625.

eTable 9. Evaluation indicators of model fit for group-based trajectory modeling after excluding all preterm births.

Model	*Shape*	*Aveep%*	*n*	$P_j\%$	$\pi_j\%$	*OCC*	E_j
Class1	Cubic	78.95	1423	23.49	23.40	12.3	0.748
Class2	Cubic	75.89	1158	19.11	20.02	12.6	
Class3	Cubic	88.26	482	7.96	8.35	82.5	
Class4	Cubic	86.85	2317	38.24	37.02	11.2	
Class5	Cubic	89.28	679	11.21	11.20	66.0	

Note: Good model fit indicated by (1)Avepp(Average posterior probability) greater than 0.7 for each group, (2)Pj(Posterior probability of group membership) greater than 5%, (3)Close correspondence Pj and πj (Probability of group membership), (4) BIC(Bayesian information criterion) close to 0, (5)The large △BIC(BIC difference between complex and simple models) the more complex models are accepted, (6)OCC(Odds of Correct Classification) greater than 5, (7)E(Relative entropy) greater than 0.7.

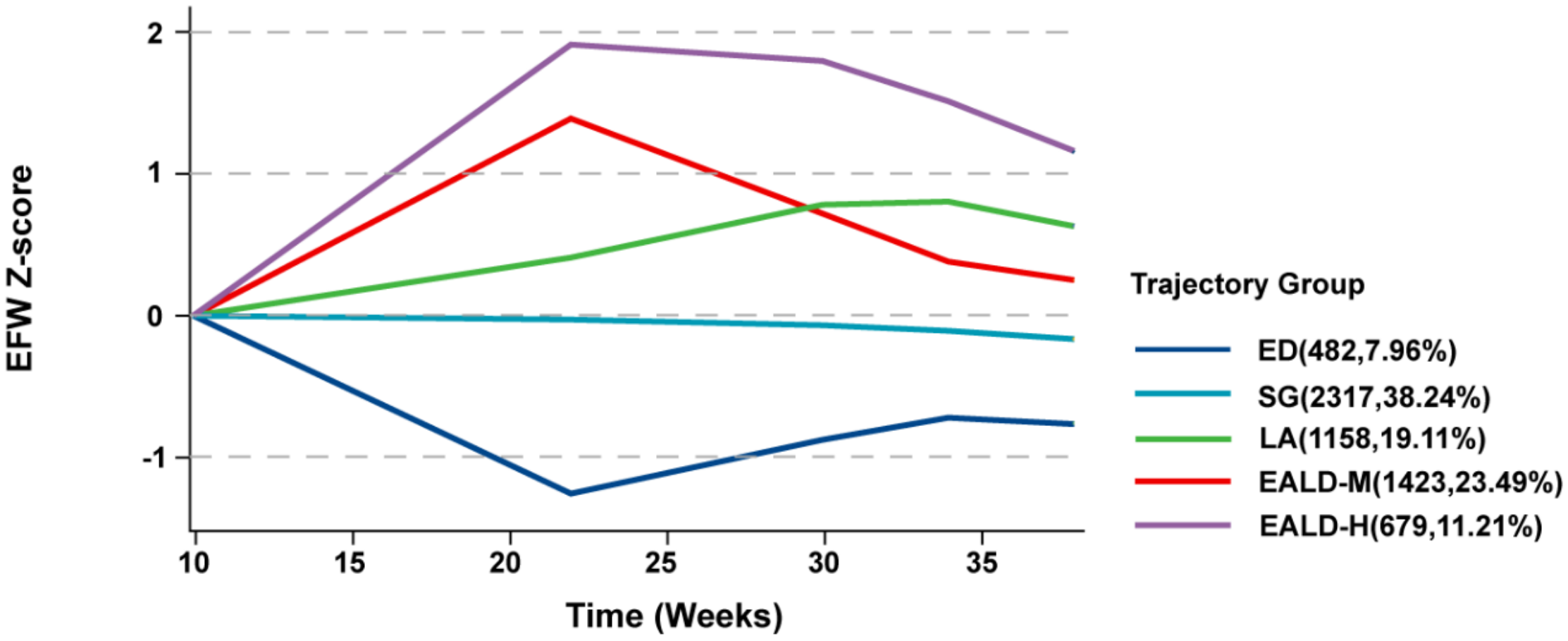

eFigure7. EFW growth patterns according to GBTM after excluding all preterm births. The EFW growth patterns in five groups derived from longitudinal records of EFW Z-scores. Group sizes are presented as percentages. Trajectory groups: ED, Early deceleration growth; SG, Stable growth; LA, Late acceleration growth; EALD-M, Early acceleration with late decline and moderate final Z-score growth; EALD-H, Early acceleration with late decline and high final Z-score growth.
